# Supplementary figures and images for: Immunotherapy does not impair ovarian function in a mouse model of breast cancer
Source: Front Reprod Health. 2026 Jul 2;8:1837989. doi: 10.3389/frph.2026.1837989 (PMC13372903; doi:10.3389/frph.2026.1837989)

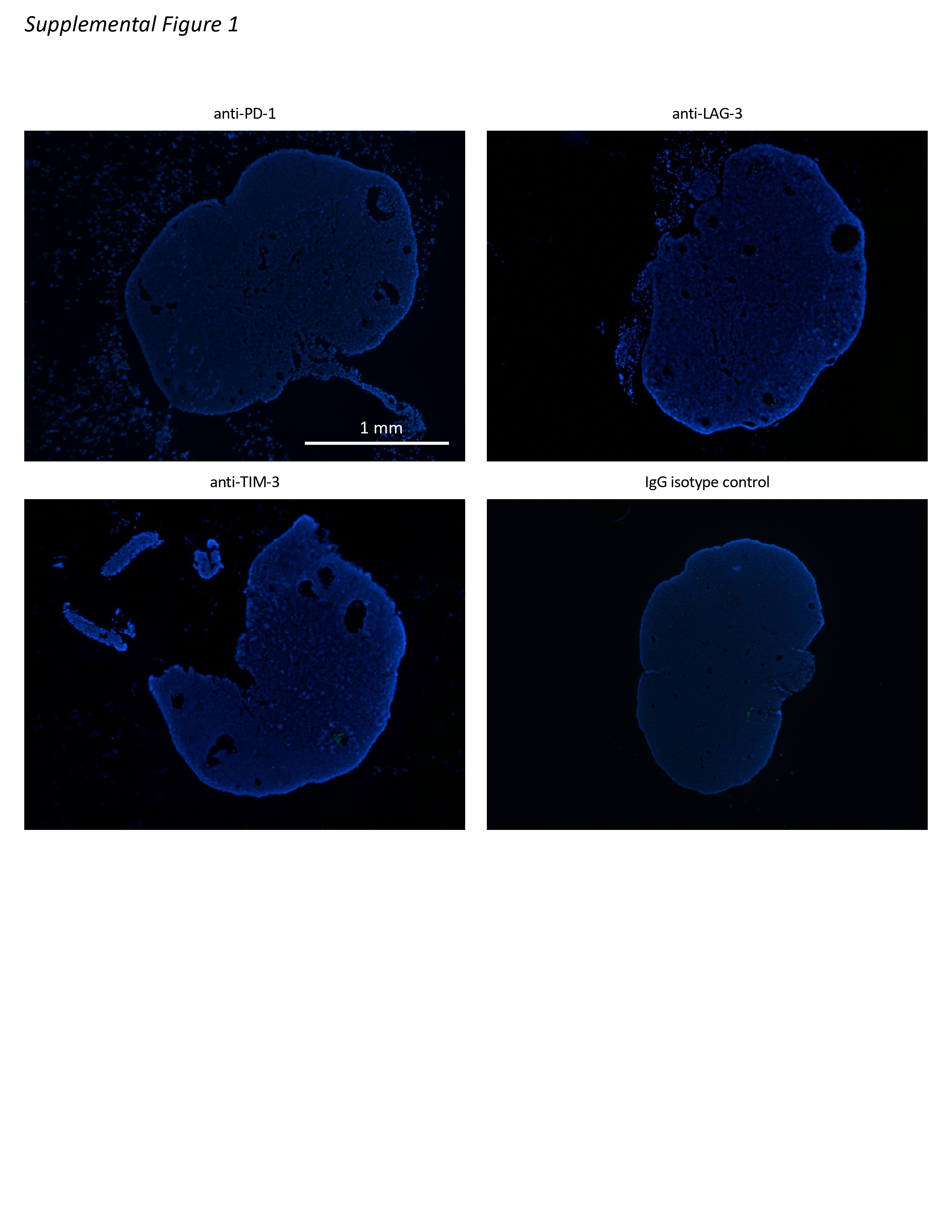

Supplement: Supplementary file 1 [file Image1.jpg]

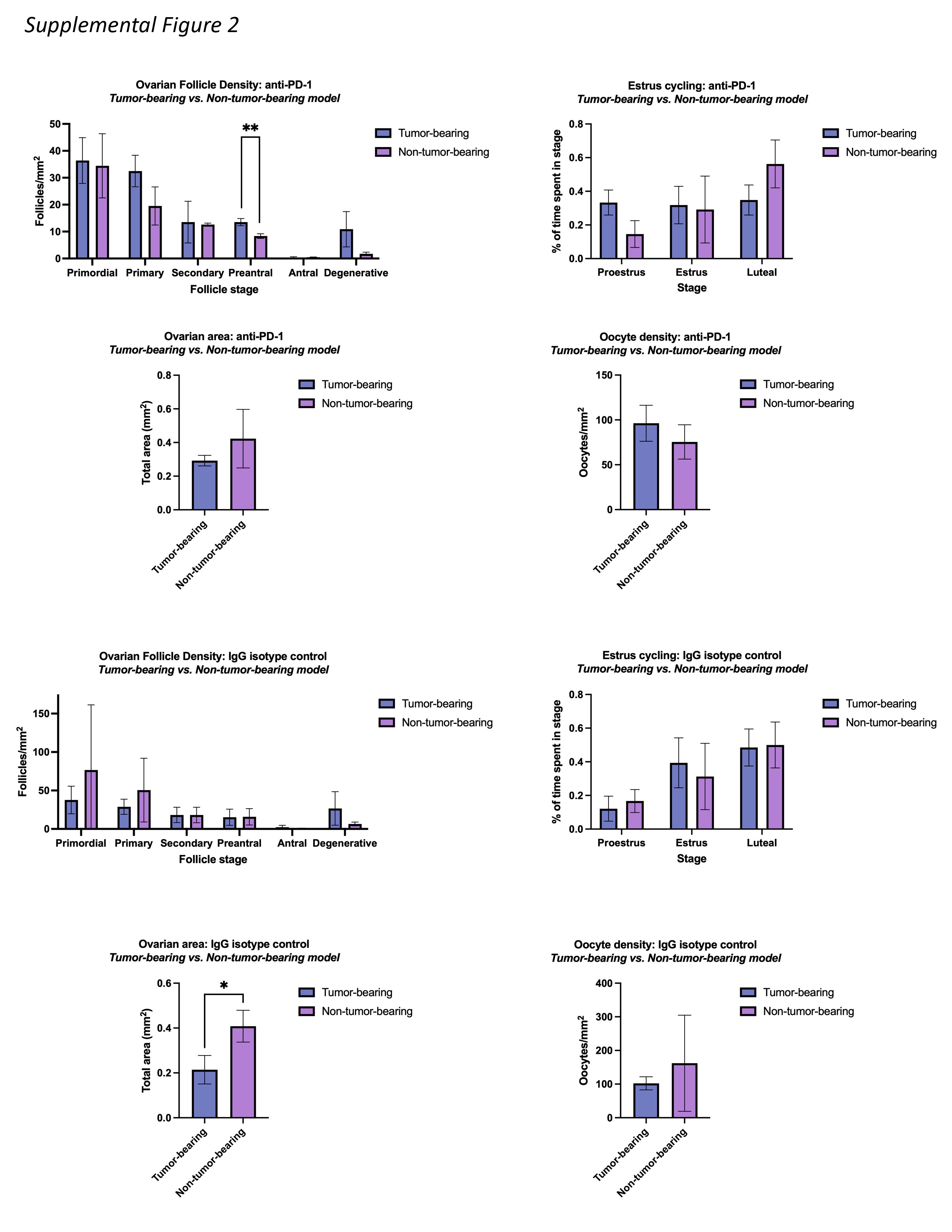

Supplement: Supplementary file 2 [file Image2.jpg]
